# COVID-19 and Emergency Department Visits: An Interrupted Time Series Analysis of Ontario and Alberta, Canada

**DOI:** 10.5811/westjem.48990

**Published:** 2026-02-27

**Authors:** Chutong Liu, Éric Lavigne, Anne Hicks, Rodrick Lim, Anna Gunz, Piotr Wilk

**Affiliations:** * Western University, Schulich School of Medicine and Dentistry, Department of Epidemiology and Biostatistics, London, Ontario, Canada; † Health Canada, Environmental Health Science and Research Bureau, Ottawa, Ontario, Canada; ‡ University of Ottawa, School of Epidemiology and Public Health, Ottawa, Ontario, Canada; § University of Alberta, Faculty of Medicine and Dentistry, Department of Pediatrics, Edmonton, Alberta, Canada; || Western University, Schulich School of Medicine and Dentistry, Department of Paediatrics, London, Ontario, Canada; # Jagiellonian University, Institute of Public Health, Department of Epidemiology and Population Studies, Kraków, Poland

## Abstract

**Introduction:**

Emergency department (ED) use declined drastically in the early stages of the COVID-19 pandemic. While the immediate effects of the pandemic are well-characterized, the longer term recovery patterns in ED use and regional differences in these patterns remain poorly understood. In Canada, provincial differences in public health policy responses may have influenced ED utilization during the pandemic, where Ontario implemented more restrictive and prolonged public health measures compared to Alberta, making Canada an ideal place to examine how regional variation in policy impacted ED use. Our objective in this study was to evaluate the impact of the pandemic on patterns of ED use in Ontario and Alberta and explore the potential differences in these patterns.

**Methods:**

Our primary outcome measure was the monthly count of all-cause ED visits in Ontario and Alberta. We obtained 146 entries of monthly counts of all-cause ED visits from April 2011–May 2023 (73,690,650 ED visits in Ontario and 27,132,554 in Alberta) from 206 EDs in Ontario and 113 EDs in Alberta and conducted a retrospective, interrupted time series analysis. Negative binomial regression models were used to estimate trends before and after the pandemic onset in March 2020 in each province and to test cross-provincial differences.

**Results:**

Ontario and Alberta experienced immediate and statistically significant reductions in monthly ED visits following the pandemic onset by 26.9% and 27.7%, respectively. Pandemic trend showed gradual recovery in both provinces. However, by May 2023 ED volumes in Ontario remained 5.5% below the expected volume, while Alberta’s exceeded it by 2.5%. Relative risk (RR) estimates confirmed significant declines in ED volumes during the pandemic in Ontario (RR = 0.64) and in Alberta (0.72). No statistically significant cross-provincial differences were observed in the immediate reduction and the speed of recovery of the ED utilization during the pandemic.

**Conclusion:**

Ontario experienced a decline in ED visits followed by a steady recovery that did not reach pre-pandemic projections, raising concern for missed care. Alberta also experienced an immediate decline but demonstrated a slightly faster recovery, eventually surpassing pre-pandemic projections. Model parameters characterizing the ED use patterns in each province were not significantly different, despite differences in provincial public health policies introduced in the pandemic’s early phases. Thus, broader national or individual level factors may have contributed more substantially to healthcare utilization than provincial policies during the COVID-19 pandemic.

## INTRODUCTION

Emergency departments (EDs) are crucial access points to healthcare, especially during times of crisis.^1^ Monitoring trends in ED utilization offers valuable insights into the overall performance, accessibility, and equity of a healthcare system.^1^,^2^ Assessment of these trends during public health emergencies like the COVID-19 pandemic is critical for informing resource distribution, managing patient flow, and identifying underserved needs.^3^ By identifying spikes and reductions in patient volume during past public health outbreaks, we can better prepare for future emergencies.^3^

In December 2019, the COVID-19 pandemic rapidly spread from country to country with the first case in Canada identified in Toronto, Ontario, on January 25, 2020.^4^ Throughout the next year, beginning in March, every province and territory in Canada entered a state of emergency and implemented public health measures to combat the spread of the SARS-CoV-2 virus. These measures included enforcing lockdowns and shifting healthcare towards virtual health.^5^

The overall timeline of policy implementation was similar across Canada, as all provinces operated under broad federal guidance on travel restrictions, vaccine procurement and distribution, and public health communication.^4^,^6^ These national measures established a common baseline of response across the country. However, notable variations existed in the specific strategies adopted by each province.^7^ In particular, Ontario and Alberta diverged in the timing and strictness of lockdowns and public health mandates, such as mask requirements and vaccine passports.^8^ Ontario adopted a more precautionary approach, implementing province-wide lockdowns and mask mandates earlier.^8^ In contrast, Alberta’s policies focused on individual responsibility, and interventions such as provincial mask mandates were not implemented until December 2020, about two months later than most other provinces.^9^ Additionally, Alberta had earlier easing of interventions in early February 2021 compared to Ontario (May 2021).^10^ These policy differences may have contributed to distinct trends in ED use during the pandemic, warranting further investigation.^11^ We hypothesized that a comparison of the trends in ED utilization between Ontario and Alberta would allow for a better understanding of how varying public health policies influence the use of emergency healthcare services during a public health crisis.^11^

Our study uniquely leveraged comprehensive ED data from Ontario and Alberta to conduct a cross-provincial comparison of all-cause ED volumes to investigate the impact of the COVID-19 pandemic on the pattern of ED visits and to assess how different public health policies affected ED volumes during the pandemic. We evaluated how the pandemic altered ED volumes by analyzing monthly provincial all-cause ED visit data between April 2011–May 2023. This time frame allowed us to better account for pre-pandemic trends and to assess whether, by the end of the pandemic period, access to emergency healthcare had been restored to its pre-pandemic levels.

**Population Health Research Capsule**

What do we already know about this issue?

*Emergency department (ED) visits dropped following COVID-19 pandemic onset, followed by a recovery to pre-pandemic levels. Ontario and Alberta had varying public health policies*.

What was the research question?

*Did ED visit recovery trends differ between Ontario and Alberta during the COVID-19 pandemic?*

What was the major finding of the study?

*No significant differences in ED decline (P = .41) and recovery (P = .13) were seen between these Canadian provinces*.

How does this improve population health?

*Policy stringency alone does not drive ED use. National factors may shape ED access, and they may be better targets to ensure equitable care in future crises*.

To achieve this goal, we used interrupted time series techniques to compare observed ED use during the pandemic with the projected trend that would have been expected had the pandemic not occurred (ie, counterfactual). We expected that generally there would have been an initial decline in ED volumes due to the implementation of lockdowns and virtual care, followed by a gradual increase towards the official end of the pandemic in May 2023, due to factors such as deferred care and loosening of public health restrictions, eventually returning to pre-pandemic levels as the healthcare system stabilized.^12^ More importantly, we hypothesized that trends in ED utilization differed between Ontario and Alberta due to variations in timing and stringency of public health policies.^9^ We predicted that Ontario’s earlier and more stringent lockdowns would lead to a faster reduction in ED volumes immediately after the onset of the pandemic, followed by a slower recovery during the pandemic,^8^,^12^ and that Alberta’s delayed interventions would result in a smaller initial decline and a more rapid return to pre-pandemic ED use levels.^8^,^11^

## METHODS

### Design

We conducted a retrospective, population-based study to analyze trends in the monthly volumes of all-cause ED visits in Ontario and Alberta before and during the COVID-19 pandemic. This analysis aimed to examine whether these volumes returned to their pre-pandemic levels by the end of the pandemic in May 2023, while accounting for pre-pandemic trends, seasonality, and population growth.

### Data

We used routinely collected administrative health data for 2011–2023 ED visits that were extracted from the National Ambulatory Care Reporting System (NACRS) database administered by the Canadian Institute for Health Information (CIHI). The ED admission data reporting to NACRS is mandated in Ontario and Alberta, and universal healthcare coverage in Canada ensures near-complete data coverage.^13^ Data were drawn from 206 and 113 EDs in Ontario and Alberta, respectively. In total, our sample consisted of 73,690,650 all-cause ED visits from Ontario and 27,132,554 visits from Alberta. We divided the ED data into two periods: pre-pandemic (April 2011–February 2020) and pandemic (March 2020–May 2023), marked by the onset of the pandemic.

### Outcome

Our outcome variable, the volume of all-cause ED visits, was defined as monthly count of these visits in Ontario and Alberta from April 2011–May 2023. To derive this variable, we aggregated the ED visit-level records from NACRS to compute the counts of all-cause ED visits for each month in each province.

### Intervention

The intervention point for the onset of the COVID-19 pandemic was March 2020 for both Ontario and Alberta, which is when both provinces declared entry into a state of emergency in response to the pandemic.^14^,^15^ We included a binary indicator to represent the onset of the pandemic in all models.

### Covariates

To account for the growing population in Ontario and Alberta between 2011–2023, we estimated the population size of each province for each month during our study period. We derived these estimates using linear interpolations based on census population counts for 2011, 2016, and 2021 and on Statistic Canada’s population projection for intercensal years.^16^ We also included a monthly time variable spanning April 2011– May 2023, which accounts for seasonal variation; seasonality was adjusted for using Fourier terms.^17^

### Statistical Analysis

Our statistical analysis closely followed the methodology outlined by Bernal et al, which has been successfully applied in different studies assessing the effects of the pandemic.^18^,^19^ First, we computed the descriptive statistics (ie, total and mean monthly count of ED visits) for ED visits during the pre-pandemic and pandemic periods, separately for each province. To estimate the effects of the pandemic on trends in the monthly volume of all-cause ED visits, we used interrupted time series^20^ segmented regression analysis.^17^,^18^ This approach estimates the expected trend (ie, the counterfactual) in the absence of the intervention (ie, the onset of the COVID-19 pandemic) and compares it to the observed trend following the intervention.^18^ By quantifying differences between these two trends, interrupted time series provides evidence of changes in the monthly count of ED visits likely attributable to the intervention.^18^

In the preliminary interrupted time series analysis, a Poisson regression model was fitted to test for overdispersion, that is, whether the variance in the monthly count of ED visits significantly exceeded its mean.^21^ Because of significant overdispersion in this model (dispersion > 1.5), we selected a negative binomial model for our interrupted time series analysis.^22^ The R code was adapted from the R package provided by Travis-Lumer et al. Specifically, we calculated the intercepts (ie, the immediate baseline level change in the monthly counts of ED visits at the intervention time) and slopes (ie, the trends in ED visit counts over time) to estimate the baseline levels and trends over time in the counts of ED visits in Alberta and Ontario. To assess the effect of the intervention and its statistical significance in each province, we computed relative risks (RR) and their corresponding 95% confidence intervals.

The RR measures the immediate change in the rate of ED visits at the start of the pandemic by comparing the level just after the intervention point to what was expected based on the pre-pandemic trend with adjustments for temporal trends, seasonality, and population size.^23^ Relative risk < 1 indicates a decrease in the volume of ED visits associated with the intervention. Following this stratified analysis for Ontario and Alberta, we assessed cross-provincial differences in the trends of ED visits using a negative binomial model with an additional interaction term for province to test whether key model parameters (ie, time trends and intervention effects) differed significantly between provinces.^24^

## RESULTS

### Descriptive Statistics

The study sample consisted of 146 counts of monthly ED visits in Ontario and Alberta, from April 2011–May 2023. In Ontario, of a total of 73,690,650 ED visits, 55,220,370 (74.9%) visits were observed during the pre-pandemic period and 18,470,280 (25.1%) visits occurred during the pandemic period. In Alberta, a total of 27,132,554 ED visits were recorded during the study period, with 20,640,306 (76.1%) during the pre-pandemic period and 6,492,248 (24.9%) during the pandemic period. On average, there were 516,078.2 ED visits and 473,596.9 visits per month during the pre-pandemic and pandemic periods, respectively, in Ontario and 192,900.1 and 166,467.9 ED visits per month, respectively, in Alberta (Table 1). In addition, as shown in the Figure, there were seasonal patterns in the volume of ED visits with the dips being the most noticeable in February and November; these results are consistent with patterns reported in ED utilization data across Canada.^25^

### Interrupted Time Series Analysis

#### Stratified Analysis

We analyzed trends in the monthly counts of ED visits during the pre-pandemic and pandemic periods by fitting negative binomial models, separately in Ontario and Alberta (Table 2). In Ontario, during the pre-pandemic period (April 2011–February 2020), the count of monthly all-cause ED visits was significantly increasing linearly (*P* < .001) with time by 0.13% visits per month from 481,350 in April 2011 to 553,114 visits in February 2020. At the onset of the pandemic in March 2020, the count decreased to 404,204 visits, representing a 26.9% reduction (Figure 1). Following this initial drop, the volume of ED visits exhibited a consistent monthly increase, with a positive slope indicating a statistically significant (*P* < .001) increase of 0.82% visits per month, reflecting the rebound in ED utilization after the sharp decline. However, despite the growth in the monthly count of ED visits during the pandemic, the monthly number of ED visits at the end of the pandemic (549,453 visits in May 2023), remained below the estimated number of ED visits per month assuming no interruption (581,234 visits), as predicted by the counterfactual. In terms of the RR, there was a statistically significant (*P* < .001) decrease in the monthly count in ED visits during the pandemic period (RR = 0.64, 95% CI, 0.59–0.68). This suggests that, on average, the count of monthly ED visits during the pandemic was 36% lower than the average count of ED visits predicted by the counterfactual, based on the pre-pandemic trend.

Figure 1 illustrates the results for the province of Alberta. The pre-pandemic trend was slightly negative but not statistically significant (−0.01% visits per month, *P* = .55), indicating that there was neither increase nor decrease in monthly ED visit counts during the pre-pandemic period (Table 2). At the beginning of the study period (April 2011), there were approximately 193,541 all-cause ED visits per month. After the onset of the pandemic, there was a decline from 192,249 visits in February 2020 to 138,936 visits in March, which represents a 27.7% reduction. Following this initial drop, the volume exhibited a statistically significant increase of 0.93% visits per month (*P* < .001). This recovery reflects a rebound in ED utilization after a sharp decline.

By the end of the pandemic period (May 2023), the observed volume of ED visits (196,541 visits per month) exceeded the number of ED visits predicted by the counterfactual (191,786 visits per month). The pandemic period showed a statistically significant (*P* < .001) decrease in the RR of monthly ED visits (RR = 0.72, 95% CI, 0.66–0.78), implying that the count of monthly ED visits during the pandemic was 28% lower than the monthly count expected without the intervention. It is important to note that although the counterfactual lines appear straight in Figure 1, minor model adjustments were applied; however, these changes were too small to be visually discernible at this scale.

#### Differences between Ontario and Alberta

The focus of this analysis was to assess statistical significance of differences between Ontario and Alberta in the key model parameters. First, Ontario’s positive slope for the pre-pandemic trend in the volume of all-cause ED visits (0.13%) was significantly different (*P* < .001) than Alberta’s slope prior to the onset of the COVID-19 pandemic (-0.01%). The results from the stratified analysis suggest that, after the onset of the pandemic, both Alberta and Ontario experienced a significant immediate decline in the monthly counts of ED visits (27.7% and 26.9%, respectively); however, the cross-province difference in these declines was not significantly significant (*P* = .41). Finally, Ontario and Alberta’s pandemic trends showed a statistically significant upward increase in monthly counts of ED visits with Ontario’s recovery trend appearing to be flatter (0.82%) than in Alberta (0.93%); however, the results from the model testing cross-provincial differences indicated that this difference was not statistically significant (*P* = .13). Overall, although Ontario had higher and faster growth in the monthly counts of all-cause ED visits before the pandemic, both provinces experienced similarly substantial declines in the volume of ED visits at the onset of the pandemic and similar recovery trends during the pandemic.

## DISCUSSION

In this study we explored trends of ED utilization in Ontario and Alberta before and during the COVID-19 pandemic. Results indicate drastic decreases in ED volumes and gradual recoveries following pandemic onset in both provinces, which support our general expectations. However, the magnitude of the decline at the onset of the pandemic and the rates of recovery during the pandemic were not significantly different between the provinces, contrary to our prediction. This indicates that Ontario’s more stringent public health measures did not lead to a larger initial decline and a slower recovery. Thus, the lack of statistically significant differences between the two provinces suggests that differences in policy stringency had a limited impact on ED utilization.

While lacking in statistically significant cross-provincial differences, this study remains important for informing on the trends and components associated with ED utilization. These non-significant findings highlight that additional factors, such as national regulations and public perspectives, may contribute to ED volumes to a greater extent, masking the effects of provincial-level policies. This poses more questions regarding population-level responses during public health interventions, calling for further research and exploration.

Similar patterns of gradual recovery observed in our analysis were also reported in other studies. As Canadian studies with ED data extending to the end of the pandemic remain limited, studies conducted in the United States may serve as a useful reference for understanding long-term trends in ED use. One of those studies found that following a significant drop in ED volume an overall rebound occurred, but volumes did not return to pre-pandemic levels by May 2023 in some states, which aligns with our estimates for Ontario.^26^ This may be due to long-term changes in healthcare delivery and utilization such as the establishment of virtual alternatives that reduced the patient’s need to visit the ED. Furthermore, provisional data from NACRS on ED visits and length of stay indicate increasing demand and persistently high wait times in Ontario EDs in 2022 and 2023, suggesting that the emergency care setting is struggling to meet population needs.^25^,^27^ These prolonged wait times have worsened post-COVID-19, further reflecting the rising demand for ED services.^27^ These conditions may deter individuals from using emergency care following pandemic onset.

In contrast, the rebound of Alberta’s ED visits to surpass counterfactual projections, based on the pre-pandemic trends, may indicate less change in the level of demand, potentially due to the less stringent and shorter public health policies implemented during the pandemic. As a result, patterns of ED utilization were less disrupted and individuals were more likely to revert to pre-pandemic behaviours once restrictions were lifted. As mentioned previously, it is also important to point out that Alberta’s monthly pre-pandemic volume of ED visits was not increasing, comparing to a positive trend in Ontario, making it easier for ED volumes to recover to the lower counterfactual levels than to the higher levels observed in Ontario.

Contrary to our findings that differences in timing and stringency of public health policies adopted in Alberta and Ontario did not impact the trends in ED volume recovery, other studies have found a negative association between the stringency of lockdown measures and ED visits.^28^ While these studies were performed in other countries, they demonstrate how public health stringency policies can influence ED use. A multicentre study in the Netherlands found a significant association between the stringency of lockdown measures and reductions in ED visits across time, with the most substantial declines occurring during periods of high stringency.^28^ Additionally, a study examining ED visits in 10 countries found that a 10% increase in mean stringency index led to a 3.3% point reduction in relative health service volume, including EDs, during the COVID-19 pandemic.^29^

Our results suggesting lack of significant differences between Alberta and Ontario may be due to the existence of national-level policies implemented in all provinces across Canada in addition to different provincial-level regulations, which may have led to more uniform changes in ED utilization and diminished the impact of regional policy differences. Furthermore, national news and publications may have influenced individuals’ behaviours consistently across Canada, regardless of their province of residency.

As the first Canadian study to conduct long-term comparative analysis of all-cause ED visits in Ontario and Alberta, it contributes new insights on healthcare utilization during the COVID-19 pandemic. Using a robust interrupted time series design and assessing the impact of provincial stringency measures on the patterns of ED volumes, we evaluated whether regional differences in public policy during the pandemic affected healthcare use.

## LIMITATIONS

Although interrupted time series analysis is a robust quasi-experimental design, it is subject to several limitations. First, we assumed that no other major intervention occurred during the pandemic that might have affected ED volumes. However, various overlapping policy changes were implemented during the pandemic, making it challenging to isolate the effect of the pandemic itself on the volume of ED visits. Consequently, the observed changes in ED utilization may reflect the cumulative impact of the pandemic alongside additional confounding factors. Second, interrupted time series requires a substantial number of time points both before and after the intervention to detect changes in trend. Fewer post-pandemic timepoints were available given the length of the pandemic, limiting the precision of the long-term trend estimates. Future research should address post-pandemic patterns using extended follow-up periods to assess long-term trends in ED utilization.

Third, while we accounted for seasonality and population growth in each province, other confounding variables—such as changes in patient acuity, health-seeking behaviour, and access to alternative care—may have influenced the observed trends. This introduces uncertainty regarding whether the changes in ED utilization were solely due to the pandemic. Incorporating interrupted time series analysis with complementary data sources, such as virtual care utilization and mortality trends, could help separate these effects.

## CONCLUSION

Both Ontario and Alberta experienced significant immediate declines and gradual recoveries in ED utilization during the COVID-19 pandemic. At the official end of the pandemic in May 2023, ED volumes in Ontario had not returned to levels projected by pre-pandemic trends; however, they returned and surpassed the predicted levels in Alberta. The observed reduction in ED volumes in Ontario raises the concern that the population is delaying necessary emergency healthcare, causing unmet needs and worse health outcomes. Despite differences in timing and stringency in public health policy introduced during the initial phase of the pandemic, the patterns of ED use were not significantly different between Alberta and Ontario, suggesting that broader national-level factors and changes in health-seeking behaviour may overshadow provincial policy effects.

Longer term trend analyses beyond the end of the pandemic are necessary to assess whether ED utilization has returned to its pre-pandemic trends or the pandemic has led to a sustained shift in emergency care usage. Population-specific (eg, age, socioeconomic status) and cause-specific assessments should be performed to help identify subgroups disproportionately affected by changes in ED utilization. These studies are critical for informing health system recovery planning to construct more resilient emergency care models for future public health crises.

## Figures and Tables

**Figure:**
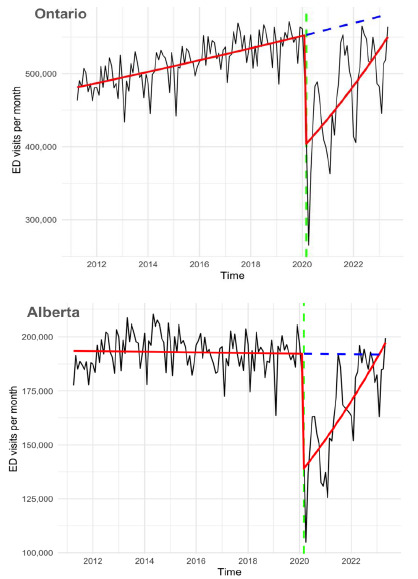
Monthly all-cause ED visits over time before and after the onset of the COVID-19 pandemic in Ontario and Alberta, Canada. The green vertical dashed line represents the intervention point (the onset of the pandemic in March 2020). The black line represents the observed monthly ED visits. The red line represents the linear trend from a negative binomial regression model adjusting for seasonality and population. The blue dashed line represents the predicted trajectory of ED visits if there was no intervention (the counterfactual). *ED*, emergency department.

**Table 1:** Descriptive statistics for all-cause emergency department visits in Ontario and Alberta during the pre-pandemic and pandemic periods.

Province	Estimate	Pre-pandemic period (April 2011 – Feb. 2020)	Pandemic period (March 2021 – May 2023)	Total (April 2011 – May 2023)
Ontario	Total visit count	55,220,370	18,470,280	73,690,650
	Monthly mean	516,078.2	473,596.9	504,730.5
Alberta	Total visit count	20,640,306	6,492,248	27,132,554
	Monthly mean	192,900.1	166,467.9	185,839.4

Source: 2011–2023 National Ambulatory Care Reporting System.

**Table 2:** Parameter estimates of the linear trend for monthly emergency department visits during the pre-pandemic and pandemic periods in Ontario and Alberta from the interrupted time series stratified analysis.

Province	Trend type	Slope (change in percentage of ED visits per month	Intercept (number of ED visits at the intervention month)	Number of ED visits at the end of the pandemic (counterfactual vs. observed)	Relative risk (95% CI)
Ontario	Pre-pandemic trend	0.13% (P < .001)	553,114	581,234	0.64 (0.59, 0.68)
	Pandemic trend	0.82% (P < .001)	404,204	549,453	
	Difference between pre-pandemic and pandemic (%)	530.8%	−26.9%	−5.5%	
Alberta	Pre-pandemic trend	−0.01% (p = .55)	192,249	191,786	0.72 (0.66, 0.78)
	Pandemic trend	0.93% (P < .001)	138,936	196,541	
	Difference between pre-pandemic and pandemic (%)	14,815.9%	−27.7%	2.5%	

Source: 2011–2023 National Ambulatory Care Reporting System.

*ED*, emergency department.
